# A Review of Digital Twinning for Rotating Machinery

**DOI:** 10.3390/s24155002

**Published:** 2024-08-02

**Authors:** Vamsi Inturi, Bidisha Ghosh, Sabareesh Geetha Rajasekharan, Vikram Pakrashi

**Affiliations:** 1 Mechanical Engineering Department, Chaitanya Bharathi Institute of Technology (A), Hyderabad 500075, India; inturivamsi_mech@cbit.ac.in; 2 Quant Group, Civil, Structural and Environmental Engineering, Trinity College Dublin, D02 PN40 Dublin, Ireland; bghosh@tcd.ie; 3 Department of Mechanical Engineering, Birla Institute of Technology & Science Pilani, Hyderabad Campus, Pilani 500078, India; sabareesh@hyderabad.bits-pilani.ac.in; 4 UCD Centre for Mechanics, Dynamical Systems and Risk Laboratory, School of Mechanical and Materials Engineering, University College Dublin, D04 V1W8 Dublin, Ireland

**Keywords:** digital twin, rotating machinery, transmission, industrial, machine learning

## Abstract

This review focuses on the definitions, modalities, applications, and performance of various aspects of digital twins (DTs) in the context of transmission and industrial machinery. In this regard, the context around Industry 4.0 and even aspirations for Industry 5.0 are discussed. The many definitions and interpretations of DTs in this domain are first summarized. Subsequently, their adoption and performance levels for rotating and industrial machineries for manufacturing and lifetime performance are observed, along with the type of validations that are available. A significant focus on integrating fundamental operations of the system and scenarios over the lifetime, with sensors and advanced machine or deep learning, along with other statistical or data-driven methods are highlighted. This review summarizes how individual aspects around DTs are extremely helpful for lifetime design, manufacturing, or decision making even when a DT can remain incomplete or limited.

## 1. Introduction

**Figure 1:**
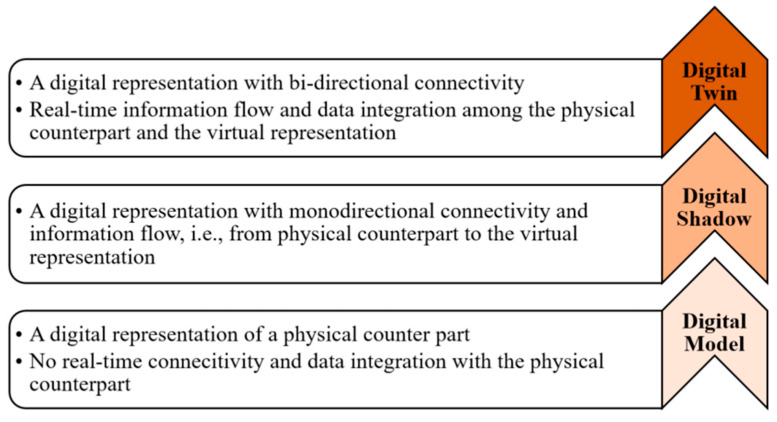
Various levels of the integration of a typical physical counterpart with its virtual copy in the virtual space—adapted from [5].

**Figure 2:**
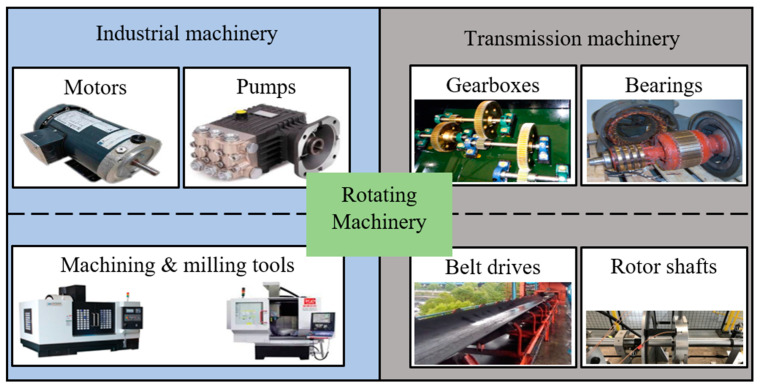
Significant rotating machinery (industrial and transmission) used in various industrial firms.

**Figure 3:**
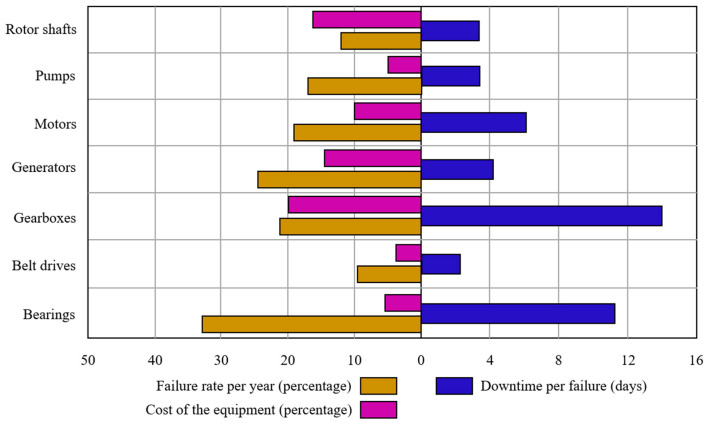
A bar chart depicting the average failure rate, cost of the equipment, and downtime of various rotating machinery of a typical off-shore wind turbine (WT) firm—adapted from [20,21].

Digital twins (DTs) are often regarded as a significant culmination towards Industry 4.0, where sophisticated digitization assists well-informed decision making. The Production Engineering Encyclopedia defines that “*The DT is a representation of an active/unique ‘product’ which can be a real device, object, component, intangible asset, service, or a system consisting of a product and its related services*” [1]. A DT is a computable virtual abstraction of a real-world entity with bi-directional real-time connectivity with its physical counterpart throughout its life cycle. Data connectivity and integration across a physical counterpart and its virtual representation can be possible via a digital model, a digital shadow, and a digital twin (refer to Figure 1) [2]. Thus, a DT is different from a finite element model as it is a digital copy of a physical system and can update its parameters simultaneously with the state of the physical entity [3]. More importantly, a DT can describe the current state of the physical entity and predict the future state. The DT replicates the physical system to predict failures and opportunities for change and prescribes real-time actions for optimizing and/or mitigating unexpected events, observing and evaluating the operating profile system [4]. With the advent of Industry 4.0 and the advances in the Internet of Things (IoT), artificial intelligence (AI), data analytics, and extended reality (XR), DTs have been applied in various fields [5]. A recent analysis states that 75% of IoT-implementing organizations currently use DTs and intend to in the upcoming year [6]. As per one of the estimates, the global DT market will reach USD 35.8 billion by 2025 from USD 10.1 billion in 2023 [7]. Among them, the predictive maintenance of rotating machinery through a DT enables precise health/condition state identification and proactive fault prognosis, thereby ultimately enhancing reliability at reduced costs [8]. Rotating machinery, such as gearboxes, motors, generators, rotor shafts, pumps, etc., are regarded as core equipment of any industry, and their maintenance is priority. Among them, gearboxes, bearings, belt drives, and rotor shafts are regarded as key components of transmission machinery and are found in various applications such as wind turbines (WTs), including automobiles and aerospace, along with industrial machinery, like motors, generators, pumps, and machine tools (cutting, milling, or drilling), for industrial production and manufacturing systems (refer to Figure 2). This industrial machinery is often integrated with other components of production/manufacturing systems to achieve improved accessibility and adaptability. Owing to complex integrations and varied operating conditions, industrial machinery often encounters unexpected malfunction, which can result in extended downtimes and poor product quality [9,10]. This transmission machinery is often subjected to volatile and harsh operating conditions, eventually prone to the nucleation of contextual anomalies (local/mono-defects), which may transform as collective anomalies (compound defects) and lead to unexpected downtimes [11,12]. For instance, the average downtime per failure, the failure rate per year of operation, and the cost of equipment of a typical wind farm industry are described in Figure 3. Thus, in order to enhance the availability, productivity, and operational safety and to reduce the downtime and the rate of unexpected failure, health/condition assessments, damage diagnostics, and the prognostics of transmission machinery are being implemented [13,14]. Condition monitoring (CM), regarded as one of the predictive maintenance tools, has received considerable attention and is being implemented prosperously in fault diagnosis and prognosis by using single/individual or collective multi-sensor information (e.g., accelerometers, acoustic sensors, or lubrication oil sensors) to perform fault detection, characterization, and the diagnosis of defects in transmission machinery [15,16,17]. Additionally, few researchers have postulated integrating/combining individual sensor information as it has the advantage of unprecedented fault detection capabilities [18,19].

Numerous systems and industries are projected to adopt DT technology to improve ubiquitous connectivity, guarantee real-time interaction with the physical counterpart, and establish intelligent automation [22]. In the context of Industry 4.0, the topic of DTs has received continued attention for health state monitoring, intelligent fault diagnosis, establishing health indicators, and fault prognosis. Studies around DTs are also popular due to their ability to replicate a wide range of facets of a product, component, object, system, process, or service. Table 1 presents a consolidated account of several prior studies reviewing DT principles, frameworks, architectures, and engineering applications. There exists diverse semantics in DTs, along with diverse patterns of their implemented practice and application context.

Physical space has played a significant role in the maintenance and decision making of industrial machinery. Additionally, Industry 4.0-related processes aim to include a range of technologies like IoT, cloud computing, AI, machine-vision, and XR to enhance real-time decision making and support automation in industrial equipment [30,31]. These technologies can often integrate physical and virtual spaces, creating possibilities for better scalability and flexibility of manufacturing systems [23,32]. For instance, IoT utilizes a wide range of sensors to acquire data from physical objects, and this collected data can be used for creating a digital model/representation of a physical object [33]. Similarly, cloud computing technologies store large volumes of data and offer an efficient and accessible data repository [34]. AI algorithms can lead to effective data analysis guided by data semantics, eventually providing insights about the status of the product or system [35], which can inform crucial information like reliability and performance evaluation estimates of manufacturing systems, influencing the product life cycle, precision, and quality [36]. Preventive maintenance is implemented in many industries to minimize the production life cycle cost by identifying anomalous or atypical behavior of the machinery via a sensor information-based detection model and related markers [37,38]. Conventional preventive fault detection methods are broadly categorized as model-based, knowledge-based, data-driven, and hybrid-based methods, respectively [8,39]. Model-based fault detection methods require an appropriate theoretical first-principles model (either mathematical or numerical) to describe the health state of the system. However, these models are often developed by considering the linear or simplified relationship among the sub-systems. It is also challenging and laborious to establish a high-fidelity theoretical model for a complex system due to it being subjected to varied operating conditions and multi-body interactions, which may be non-linear and non-trivial to deal with, and often even include stochasticity [39,40]. A knowledge-based fault detection method usually forms a set of rules through a human expert’s knowledge. These methods are often appropriate when a theoretical/fundamental model is not available and are helpful in diagnosing pre-defined faults but hardly suitable for detecting undefined/anonymous faults [41]. On the other hand, the prolific implementation of sensors, data acquisition devices, IoT, and data analytics can allow large amounts of data to be captured and monitored from the physical system. Data-driven fault detection methods (either statistical or non-statistical) with appropriate data analyses can unveil hidden patterns contained in data, and they are becoming particularly popular [42]. This approach can often better accommodate non-linearity and stochasticity in both the system and the signal, respond to complex systems in an improved manner, and provide a way to update and augment first-principles-based mathematical models. However, such models require large datasets for the accurate analysis and development of models adapted for updating with periodic or real-time data availability [43,44]. Since each method has distinct advantages and limitations, many researchers have proposed hybrid fault detection methods by combining physical and data-driven models. The physical model-based approach offers interdisciplinary/multi-disciplinary coupling and addresses the unavailability of data samples for each scenario, whereas a data-driven approach has the scope of optimizing physical models to create prediction models with an improved accuracy. Combining individual approaches into a hybrid approach offers extended potential in promoting the core visions of Industry 4.0 [45,46]. The DT concept accommodates the hybrid fault detection approach. Since a DT offers better scalability (the ability to provide insights at various scales ranging from small to large), interoperability (the ability to establish equivalence among various models), expansibility (the ability to integrate internally as well as externally), and fidelity (the ability to represent the actual behavior of the physical counterpart), it can accomplish intelligent fault diagnosis, eventually substantiating the core objectives of Industry 4.0 [26,47,48]. Additionally, a DT has the scope of assisting some core principles of Industry 5.0, i.e., human-centricity (by adapting to novel technologies which are capable of enhancing human safety by avoiding catastrophic failures) and sustainability (by simulating the data for the unavailable/invisible operating scenarios/combinations), thus minimizing the need to perform numerous experiments and process materials, eventually leading to more sustainable manufacturing [49,50].

**Figure 4:**
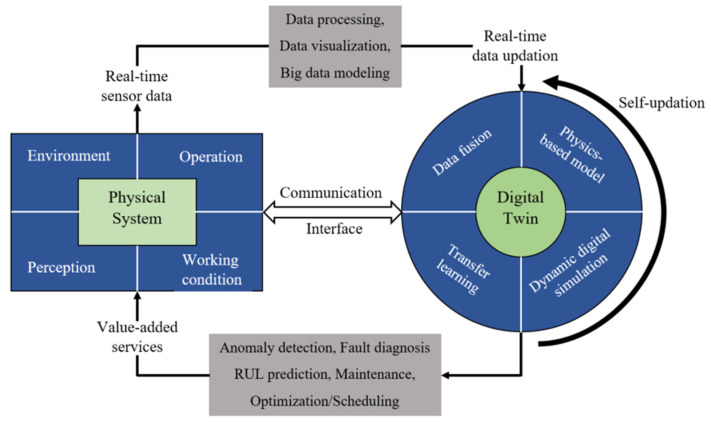
Equivalent representation of general architecture of digital twin.

A DT contains a set of adaptive models that can emulate the behavior of a physical system in a virtual system, obtaining real-time data to update itself along its life cycle [51]. There are static and dynamic features of a DT; the static feature of a DT is capable of approximating the behavior of the physical system during the simulation, while the dynamic feature of a DT is capable of duplicating and imitating the actual behavior of the physical system during emulation [52,53]. Also, during the manufacturing phase, a DT enables simulations to periodically analyze the interactive behavior among the production entities of a physical system [26]. During the service phase, a DT often performs health state monitoring and predicts the life of the components of the physical system. Thus, a DT needs to evolve synchronously with the physical system along its whole life cycle within time periods of relevance, modify its initial configuration, and adapt to the current situation. This leads to better monitoring performance metrics, predicting future performance and remaining service life, and making replacement or rehabilitation decisions by aligning to available data (Figure 4). Additionally, a DT integrates multi-disciplinary (modeling various mechanisms simultaneously), multi-physical variables (modeling various physical disciplines parameters simultaneously), multi-scale (modeling at various instances of time and/or frequency), and multi-probability (modeling various independent events/occurrences) by entirely using the physical model, sensor updating, and historical operation data [54,55].

It is observed from the existing literature that while there exist reviews of DTs in several fields, like manufacturing systems [23,24], healthcare systems [5,24,26], aerospace systems [5,29,30], maritime [26,29], electromechanical systems [28], industrial applications [22,24,42], civil infrastructure [42], and rolling bearings [28], there is a gap in terms of such a review for providing a comprehensive analysis of DT implementation approaches for rotating machinery. This paper attempts to address the gap by presenting some of the salient features of DTs in rotating machinery along with some relevant possibilities, challenges, and limitations of DT implementation and its use in rotating machinery. Considering the application characteristics of DT technology for rotating machinery, the specificity caused by different development levels and application environments and the evolution focus throughout various phases are emphasized. Our research examines perspectives and narratives of a future agenda for researcher scholars based on real-time applications available in the literature. This paper first starts with the definition of a DT, along with the advantages and various modalities and tools used for its construction, and subsequently moves to the roles and functions of IoT, AI, and data analytics in a DT. Some research challenges and future prospects in high-fidelity DT construction, data fusion, multi-physics coupling, ML, and cloud-edge collaboration are discussed next, including detecting and characterizing events or features of interest (anomaly detection, degradation prediction, etc.) in rotating machinery. This review thus presents the current state of the art on DTs and rotating machinery, while also assimilating potential advantages and challenges for ongoing developments in the sector, in the context of a wide range of applications around Industry 4.0, providing an eventual pathway for guidance around efficacy, accuracy, and errors.

## 2. Digital Twin

### 2.1. Definition and Viewpoints of Digital Twins

**Figure 5:**
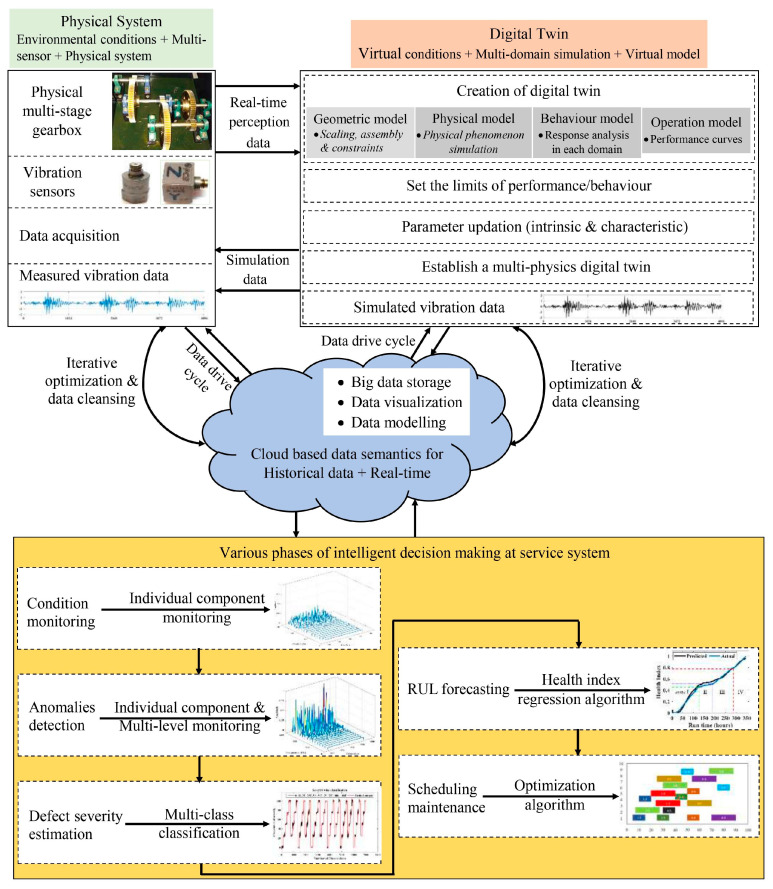
An illustration of a DT-aided intelligent decision making framework for accomplishing the predictive maintenance (health state monitoring, fault diagnostics, and prognostics) of a typical gearbox system [65,66].

DTs are most particularly valuable when a component/system is subjected to change over time, thus rendering its initial working state invalid, and when the measured data signals that can be correlated with the change in working state can be visualized. According to Industry 4.0, a *DT uses the physical model, sensor updates, historical operational data, integrated multi-scale and multi-probability simulations, multi-physical, multi-disciplinary phenomenon, and complete mapping in a virtual space for reflecting the entire life cycle process of the corresponding physical system* [56]. Thus, a DT is a high-fidelity, multi-physics, multi-scale, multi-domain, probabilistic simulation of a physical system that uses the available physical laws, sensor updates, and historical data to emulate the behavior of the physical system virtually [46,57]. It also has the possibility of performing data mining to detect existing defects and to forecast the upcoming events of interest with an appropriate synchronization rate [42,58]. Therefore, a DT is used for a wide range of scenarios, and the distinction across each field must be made clear. Table 2 describes DTs’ viewpoints concerning the scenario of an industrial firm. A typical architecture of a DT-assisted maintenance framework contains three vital ingredients: the physical space, virtual/digital space, and data flow cycle (refer to Figure 5). The DT replicates the behavior (fully or partially) of the corresponding physical system in the virtual space by generating the same output as the physical system on given input values. The data flow cycle feeds the data from a physical system to its DT and takes back the information and process from the DT to the physical system. Furthermore, integrating data semantics and big data analytics through machine learning (ML) algorithms enriches the research potential with new opportunities such as constant and remote monitoring and intelligent decision making.

**Table 2:** The viewpoints of digital twins in various scenarios.

Scenarios	Viewpoints	Ref.
Design	Realize system reconfiguration and redevelopment and improve research and development efficiencies	[48]
Production	Realize the autonomy of systems’ production processes and visualization, make the process flexible, enable quality assurance, and improve efficiency	[59,60]
Operation	Monitor the system’s installation and system usage, enhance the control of the system, and realize optimal operation	[61,62]
Maintenance	Monitor the current state of the system, the configuration of data, fault diagnostics, and prognostics	[63,64]

### 2.2. Modalities of Digital Twins

**Figure 6:**
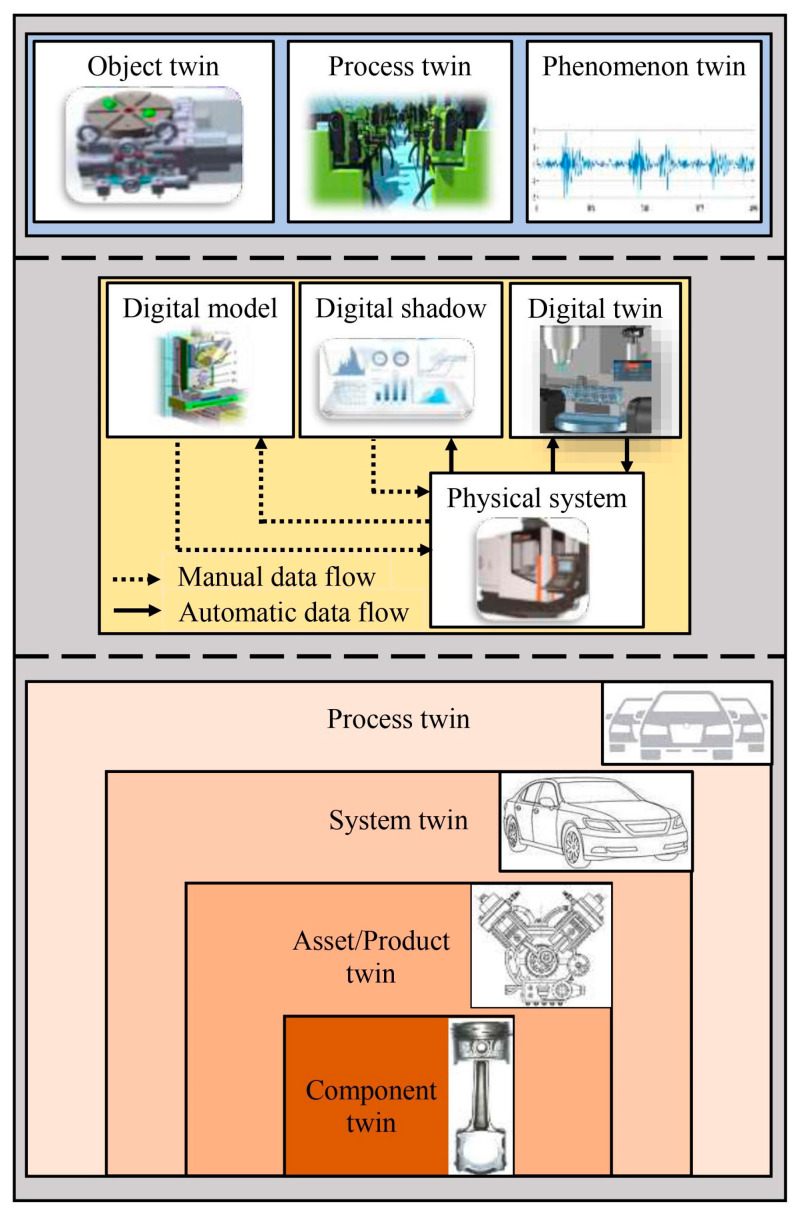
Digital twin modalities in different avenues (bottom-up approach). Various modality levels of implementation of digital twin for typical car manufacturing system [69]; various levels of integration for CNC machine–physical system, digital model, digital shadow, and digital twin [68]; various autonomies of DTs for typical machining job shop describing object twin (machine tool), process twin (job shop), and phenomenon twin (tool vibration) [57,71].

Various nomenclatures, such as a digital model, digital shadow, and digital twin, exist in industrial practice (refer to Figure 6). The digital model (DM) is a comprehensive representation of the physical system under investigation in the digital space but does not communicate with the physical system, and real-time connectivity and automatic data exchange are hardly possible [67]. They include analytical, numerical, and simulation models. Next, a digital shadow (DS) ensures one-directional connectivity between the physical system and the digital counterpart so that any modification (operational and/or geometrical) in the physical system’s state would lead to the modification of the digital counterpart’s state in the virtual space [2]. Thus, a DS establishes a unidirectional real-time automatic data exchange and enables heterogeneous sensor data acquisition, management, and analysis. Therefore, the DS is a prerequisite for applying techniques and models of data analysis and evaluation. In contrast, a DT contains all of the information derived from modeling (DM) and the data acquired during the real-time operations (DS), i.e., a bi-directional real-time data exchange is achieved [68]. Also, various levels of implementation are possible for a DT, including a component twin, asset/product twin, system twin, and process twin (Figure 6) [69]. Typically, there are three types of digital twins: an object twin, process twin, and phenomenon twin [70]. The object twin is the digital twin of a real-world object (e.g., a machine tool, work piece, cutting tool, etc.) used in a given manufacturing environment. Additionally, a process twin is the digital twin of a process sequence in a given manufacturing environment. Finally, a phenomenon twin is the digital twin of a machining phenomenon (e.g., tool wear, surface roughness, cutting force, etc.) in a given manufacturing environment (refer to Figure 6).

### 2.3. Visions and Advantages of Digital Twins

Being the high-fidelity, multi-physics, multi-scale, and multi-domain model, a DT should align to a number characteristics: (i) the DT needs to be an identical version of its physical system with a high degree of accuracy, and it needs to produce simulation data of the digital counterpart that is close to the actual physical system; (ii) the DT needs to replicate all geometric (shape, size, etc.) and physical properties (dynamics, thermal, etc.) of its physical system at multiple scales; (iii) the DT is expected to seamlessly interact with historical sensor data as well as the dynamic, real-time sensor signatures; (iv) the DT should handle semantically annotated datasets with minimal latency; (v) through heterogeneous sources of knowledge, the DT must use machine learning to obtain the required knowledge from the historical sensor signal datasets, which can be further utilized for “what-if” analyses and optimization activities [72,73]. Implementing DT-based solutions into an industrial firm may lead to several advantages, as indicated below:

- **Re-design and Interoperability**: The performance can be predicted as the DT establishes a successful connection with its physical counterpart throughout its life cycle. This enables the engineers to re-evaluate the assumptions involved so that the customization of the product/strategy can be possible [74].
- **State Monitoring and Fault Diagnosis**: A DT assists in intelligent fault diagnosis by generating simulation data for the physical system’s unavailable/invisible defect scenarios, which have complex structures, multiple parts, and intricate mechanisms [75].
- **Fault Prognosis and Optimized Maintenance**: Through a DT, the simulated data pertaining to varied operating conditions, degradation mechanisms, and failure modes can be generated, thereby saving much of the cost invested in obtaining the labeled data [46]. Thus, a DT offers the most optimal maintenance strategy by simulating various scenarios.
- **Cost-effectiveness**: With a DT, the components under investigation can be tested in various operating conditions, and damage scenarios can be simulated without incurring additional costs. Thus, without the need for expensive and time-consuming physical mock-ups, a DT allows for the efficient prediction of the effects of processes and product development on the system’s behavior [76,77].
- **Accessibility and Fidelity**: A DT provides remote and edge-based solutions by establishing a cyber-physical system that implements high-level cognitive tasks, such as data conversion, data analytics, and self-adoption [78]. In situations where local access is restricted, such as during the COVID-19 pandemic when governments enforced lockdowns, working/monitoring remotely or non-contact is the only practical option.
- **Safety and Scalability**: Owing to its predictive nature and ability to access its physical counterpart remotely, a DT can help lower the likelihood of hazardous failures and accidents in industries like the mining and oil and gas industries, where working conditions can be hazardous. In light of the global COVID-19 outbreak, nearly one-third of businesses are adopting DTs to improve employee and customer safety through remote monitoring [79,80].
- **Sustainability and Disposability**: Waste is greatly decreased when product or system prototypes are tested and simulated in a virtual environment using DT. Before a product is manufactured, prototypes can be virtually tested in various conditions to ensure the final design is perfect. This lowers development costs and time to market and prevents material waste [27].

Owing to these advantages, several industries have adopted DTs, with ever-expanding application fields including condition/health monitoring, performance forecasting, fault diagnostics, and prognostics (Table 3). A DT has the scope of enhancing the accuracy of machining operations by the real-time CM of a machine tool’s status and providing optimized operating conditions, eventually leading to a better product quality [68]. In the context of Industry 4.0, DTs have become crucial for industrial automation, aiming to comprehensively depict the system/product behavior and their associated processes [81]. With the incorporation of IoT and data analytics, it is possible to understand the system’s interaction and their real-time behavior virtually, which minimizes human interaction and optimizes the safety of the workers [22]. Additionally, DTs provide unique ideas to assist the Industry 5.0 concept by enhancing the efficiency of the production/manufacturing system and the quality of the products, decreasing material waste, and taking care of environmental concerns, thus yielding more sustainable and eco-friendly industrial firms.

## 3. Implementation of Digital Twin in Transmission Machinery

DT-based solutions have been adopted to monitor the working conditions/states of transmission machinery (gearboxes, bearings, rotor shafts, belt drives, etc.) and unacceptable deviations from ideal working circumstances to establish intelligent maintenance strategies. Wang et al. [82] constructed an analytical DT model of a rotor test rig to demonstrate its effectiveness in emulating the rotor imbalance and analyzed the amplitude of vibration signals for the quantification and localization of an imbalance defect. A parameter sensitivity analysis for characteristic variables (critical speed and imbalanced vibration amplitude) was carried out to improve the adaptability of these DT models. Chakraborty and Adhikari [83] facilitated physics-driven DT model data to an ML model for predicting the temporal evolution of system parameters (stiffness, mass, etc.) at multiples scales of a single degree-of-freedom (DOF) dynamical system. Garg et al. [84] proposed an ML-assisted DT framework for parameter estimation (stiffness, force, and acceleration) of stochastic non-linear multi-DOF dynamical systems (2-DOF and 7-DOF oscillator systems). Ritto and Rochinha [85] channeled data simulated through lumped-parameter models to ML classifiers for quantifying uncertainties in the simulated data followed by damage detection and severity prediction of structural damage. Wang et al. [86] achieved real-time monitoring of offshore WT support structures through a DT along with their reliability analysis and optimized the operation. Yu et al. [87] constructed a DT model based on a non-parametric Bayesian network for realizing the health state evolution and further implemented ML algorithms for predicting the propagation of the dynamic degradation process of a complex system. Fedorko et al. [88] proposed a numerical finite element DT model of rubber–textile conveyor belts and realized the combination of critical parameters (contact forces, thickness, and strength) causing conveyor belt drift. Moghadam and Nejad [13] established a high-fidelity and reduced-order DT model based on torsional moments of the floating WT drivetrain components. From the estimation of the critical parameters (load, stress, stiffness, etc.), the authors constructed a stochastic physics-based degradation model for the accurate monitoring of the RUL of the drivetrain main shaft. Duan et al. [89] developed a rotor blade system DT model and two-way mapping between the DT model and the physical test rig to visualize the operating condition of the whole test rig system while it is subjected to dynamic working conditions.

Liu et al. [90] proposed a DT model for predicting the RUL of roller bearing in which a phenomenological vibration model is introduced to generate simulated fault data, and a DL network is developed to achieve domain invariance of simulated data and measured data. Piltan and Kim [91] proposed an analytical DT model, and the simulated dynamic response is subjected to feature extraction and threshold construction via ML algorithms for detecting the bearing defects, and they achieved classification accuracies of 97.3%, 98.3%, and 98.3% respectively, while discriminating among the roller, inner race, and outer race defects. Farhat et al. [63] developed a lumped-parameter-based DT model and simulated the vibration response of ball bearings followed by successful validation (time-domain and frequency-domain) with the experimentally acquired vibration response through accelerometers. Furthermore, they extracted various statistical features from the simulated vibration response and provided them as inputs to ML algorithms, achieving outer race defect severity classification with an accuracy of 84%. Zhao et al. [92] established a numerical DT model of rolling bearings in ANSYS and implemented an improved generative adversarial network approach for mapping the simulated vibration response of the DT model with the experimentally acquired response. Furthermore, they fused the virtual space (DT model) information with the transfer learning approach (long short-term memory networks) for overcoming small sample or data shortage issues and achieved an RUL prediction of the main bearing of the direct-drive WT with an accuracy of ~85%. Li et al. [93] realized multi-scale fault evolution in rolling bearings subjected to a stationary operating speed through their analytical-based DT fault extension model by mapping the relationship between the bearing outer race defect width with the root mean square (RMS) value and the bearing outer race defect depth with the characteristic frequencies of the simulated vibration response. Regis et al. [94] proposed two DTs (statistics-based and physics-based) for simulating the vibration response of a bush bearing and analyzed the wear depth, wear rate, and the standard deviation parameters to estimate the wear progression of the bearing subjected to dynamic loads. The authors carried out early wear prediction through the statistics-based DT, whereas a physics-based DT yields the detection of the nucleation of uncharacteristic events followed by the prediction of wear. Zhang et al. [95] established an analytical DT model of a rolling bearing and simulated the dynamic response to achieve intelligent fault diagnostics. They demonstrated the implementation of a DL-based domain adoption algorithm to accomplish feature learning from the labeled datasets of the simulated response, eventually realizing the damage diagnosis with the acquired unlabeled vibration response about unknown damage scenarios. Shi et al. [96] proposed a dynamic DT model for achieving the local defect (on outer race) extension of a rolling bearing. Initially, the simulated vibration response from the DT model was subjected to a signal approximation approach for dynamic updating and mapping with real-time sensor data to simulate the outer race defect sizes.

Xiangjun et al. [97] established a multi-body dynamics simulation DT model of a three-stage WT planetary gearbox with an overall gear ratio of 118 and investigated the simulated vibration response through data-driven algorithms for discriminating across various defects of the gear tooth. Hu et al. [98] proposed a DT-based concept model of a gearbox and claimed that the proposed DT model can reveal the gearbox’s failure mechanisms and dynamic characteristics. Fahim et al. [99] proposed a concept using a cloud-based DT architecture with assistance from the 5G next-generation radio access network for creating a predictive model of a WT gearbox with an end goal of digital health monitoring. Zhu et al. [100] built a multi-body dynamics numerical model of the WT planetary gearbox in ADAMS and exported it to establish a simulation DT model in MATLAB to simulate the vibration response. Time-domain validation extracted the features and the characteristic frequencies to detect the gear tooth combined defect (missing teeth and pitting) and related diagnostics of the planetary gearbox. Mehlan et al. [101] developed a bond graph-based DT model in SIMPACK for the one-speed stage (high-speed) of the WT gearbox subjected to stationary operating conditions and accomplished real-time load sensing within the DT framework. Yu et al. [102] constructed a numerical physics-based DT model in MATLAB based on the Hertzian contact theory and finite elements. They simulated the vibration response for a planetary gearbox with time-domain validation via vibration data followed by statistical feature matching. The characteristic frequencies are examined in both signals’ envelope spectra to identify gearbox anomalies. Wang et al. [103] built a numerical DT model in Unity3D, establishing virtual–real communication, and simulated the vibration response for a WT planetary gearbox. The simulated vibration signals were decomposed to extract statistical features of interest and subsequently subjected to ML-based classification for discriminating amongst the various health states of the gearbox. Feng et al. [104] developed a physics-based dynamic DT model of a single-stage gearbox in MATLAB to reflect its operational status and, eventually, to monitor gearbox wear propagation. They implemented a DL-based domain transfer learning algorithm to link dynamic responses simulated from the DT model with the physical measurements and assessed the severity of gear tooth degradation that occurs through surface pitting and tooth profile change. Table 4 depicts a brief overview of different DT solutions that have been implemented for the health state monitoring, fault diagnosis, and prognosis of various transmission machinery. While DT-based strategies have shown promising results in assessing the intelligent maintenance of various transmission machinery, the widespread adoption and reliability of this technology in various industrial firms for future innovations is yet to happen.

## 4. Implementation of Digital Twin in Industrial Machinery

Industrial machinery like motors, pumps, generators, and cutting tools (machines and milling tools) are often used for energy exploitation and industrial production, where they are subjected to extreme loads, events, and operating conditions, affecting both their integrity and performance, reduction in service life, and eventual failure [111,112,113]. To address efficient utilization, safety, and reliability, health state monitoring has been implemented so that alterations in intrinsic process parameters under specified operating conditions can be analyzed to provide warnings of abnormal events at nascent states [114,115,116]. Bouzid et al. [117] proposed a novel DT model for an induction motor by combining a numerical model built through FLUX2D with a simulation model built using Simulink. The simulated signals (voltage, current, and angular velocity) were validated with real-time-measured signals for an induction motor with seven circuits. Xia et al. [78] used DT- and DL-based transfer learning approaches for the fault diagnosis of a triplex pump, where a simulation model was used to generate datasets of defect scenarios. Statistical indicators computed from the simulated and measured pressure signals with DL algorithms led to the detection of health state discrimination of defects with an overall accuracy of ~93%. Dos Santos et al. [118] used current and force sensor signals in a high-fidelity, numerical, and phenomenological DT model, where they analyzed the thermo-magnetic behavior to predict the health state of an induction motor with an error of ~4%. Huang et al. [119] built a simulation DT model in Simulink to establish virtual–real communication and simulated the phase current signal vibration responses for a permanent magnet synchronous motor. Furthermore, the simulated current signals were decomposed to extract statistical discriminators, which were then subjected to ML-based classification for discriminating the different health states of the motor. Wang et al. [120] constructed a simulation DT model of a triplex pump in MATLAB Simscape to reflect its operational status, along with intelligent fault diagnosis via a DL-based adaptive adversarial network algorithm. Information on the dynamic torque response simulated from the DT model with physical measurements were linked to discriminate between healthy and defective classes of triplex pumps subjected to various working conditions. Xia et al. [73] established a multi-physics numerical DT model of an induction motor in COMSOL for creating the datasets of label-scarce defect scenarios to assist in fault diagnosis. The simulated and acquired motor current signals were transformed to gray-scale images and subjected to ML-based semi-supervised fault diagnostics with an accuracy of ~92%. Kohtz et al. [121] simulated the motor current signals from the numerical DT model built using the FLUX software [122] pertaining to various health state scenarios of an AC motor. After successful validation with the measured currents, the signals were supplied as inputs to a KNN classifier for defect detection and classification with fewer sensors.

Wang et al. [123] developed a high-fidelity parametric DT model by considering the non-linear multi-body dynamical interactions of a five-axis drilling machine tool. They estimated the tracking error, motor current, and vibration excitation induced due to the alterations in the cutting force and tool vibrations. Ghosh et al. [124] contributed to the development of two modular architectures for the construction (DTC) and adoption (DTA) of DTs and examined the simulated torque signals to ensure the ability of milling tools for performing the intelligent health state monitoring autonomously. Luo et al. [36] proposed a multi-physics and multi-domain DT model assisted with data-driven algorithms for reflecting the actual operating scenarios, followed by an accurate RUL prediction of a CNC machine tool while simultaneously considering multi-physics indicators like thermal, mechanical, and electrical measurements. Ladj et al. [68] carried out health state monitoring followed by failure detection of a CNC machine tool of the aircraft manufacturing industry by analyzing the torque signals simulated through the functional DS model constructed in the Java^TM^ environment assisted with Gaussian mixture models. Zhu et al. [125] proposed a DT-driven framework, which integrates a numerical DT model of a work piece and a machine tool for a five-axis CNC milling machine for optimizing the machining process, achieving reduced deformations (~30%) and preparation time and improved process monitoring. Yang et al. [57] established a DT-driven hybrid approach where the simulated mechanistic model data and the real-time data are fused to predict the performance degradation in the transmission unit (worm gears) of the CNC machine tool. Initially, the numerical virtual DT model of the transmission unit was modeled, and the parameters (geometrical, physical, and operational) were tuned to achieve multi-domain simulation. Eventually, model updating was achieved through the mapping interface to simulate the contact stress signals. Wei et al. [126] constructed a multi-domain numerical DT model of a CNC machine tool by necessitating mechanical, electrical, control, and hydraulic sub-systems. Upon the estimation of intrinsic parameters (cutting force, vibration, spindle current, acoustic emission, etc.), these multi-variables are supplied as inputs to the DL-based transfer learning (LSTM networks) algorithm for modeling cutting tool life prediction with an accuracy of ~87%. Xue et al. [127] established a multi-domain phenomenological DT model through Modelica by coupling various sub-systems, including mechanical, electrical control, and heat transfer, for the in-process fault diagnostics of a CNC machine tool. The simulated axial stiffness and radial stiffness values and the acquired characteristic parameters (force, displacement, temperature, etc.) were supplied to the ML algorithm (decision tree) to assess the machine tool’s deterioration during its operation. Wang et al. [128] coupled mechanical and electrical control and constructed a multi-domain simulation DT model of a CNC machine tool spindle through Modelica. A coupling interface across the complex mechanisms and real-time virtual mapping between the DT and its physical system (machine tool spindle) were established for a stationary operating speed. Guo et al. [129] established a physics-based analytical DT model of a CNC machine tool and simulated the dynamic response signals. Upon successful validation with the measured real-time static, dynamic, temperature, and perception information of the physical system, the probability distribution function was derived for predicting the RUL of the spindle, enabling a stable manufacturing process. Additionally, several researchers have exploited the features of DTs to optimize scheduling activities of an industrial firm. Liu et al. [130] established a super network model of process machine tools and achieved intelligent scheduling. Li et al. [71] proposed a DT thread framework for anomaly detection and the dynamic scheduling of a flexible job shop by reducing the deviation between the initial and execution plans. Table 5 depicts a brief overview of different DT solutions that have been implemented for the health monitoring, fault diagnosis, and prognosis of various industrial machinery. While DT-based strategies show promising results in the in-process maintenance of various industrial machinery, manufacturing industries continue to value characteristic parameter identification, damage analysis, and machinery anomaly detection with maximum detail and early warnings.

## 5. Discussion and Conclusions

A physical system’s DT can be constructed to analyze the behavior virtually, and the computed data are helpful for remote monitoring followed by timely decision making or production engaged with [134]. However, the DT-driven health/state monitoring of rotating machinery is yet to mature, and this review identifies some technical challenges which can support better fault diagnostics:

- **Multi-physics coupling**: High-fidelity DT models simulating operating conditions and establishing the interactions between seemingly independent parameters with the interactions across various sub-systems requires a strong multi-physics coupling strategy. This multi-physics approach requires extensive understanding of the fundamental processes and their interactions and guidance of the choice of intrinsic parameters and their ranges of values (e.g., dynamics, mechanical, thermal, structural, and electrical) to obtain coherent simulated responses, leading to more realistic and useful DTs for the entire physical system [135,136].
- **Interoperability twinning**: A DT is often created by focusing on a single physical asset in a typical manufacturing firm. However, system-level twinning via interoperability twinning ensures that all individual components and/or sub-systems are integrated, and this aggregated DT provides more comprehensive and accurate insights about the system and yields an improved service [137,138]. Consequently, more examples and guidance around interoperability are strong needs.
- **Edge computing**: Real-time feedback is helpful for several DT applications, but the latency caused by long-distance data transfer impedes performance. Raw data acquired with various sensors are often heterogeneous, impacting the communication between data centers. Raw data cleansing and handling at the edge before transferring to cloud-based data processing can enhance the performance of the DT network by reducing the latency of data transmission. A DT-driven framework assisted by edge or even cloud computing can lead to a significant reduction in data distribution discrepancy while enabling high-frequency and dynamic data storing, transmission, and processing [139,140]. Obtaining data from reliable sources and displaying them in a virtual environment helps identify deviations and/or anomalies (single and multiple defects) of components and related decision making.
- **Lack of regulations**: A standardized industry-wide data transfer framework is still not realized, and an improved regulatory framework encompassing efficient data flow can enhance accessibility without sacrificing security protocols. Inadequate communication protocols and data collection standards can compromise the quality of the data being computed by the DT, which impacts the overall performance. A standardized framework (ISO 23247) is being developed to offer guidelines, strategies, and evaluation methods for creating and executing DTs in the manufacturing industry [141,142].
- **Data safety issues**: Data silos might result from improper policies governing data exchange externally (e.g., stakeholders across the industrial firm) and internally (e.g., within an organization). Also, data interoperability across various DTs needs to be examined. There are also concerns about ownership, privacy, transparency, confidentiality, and the security of the data computed [5]. Industrial firms are required to install intelligent security measures to protect the sensitive information of the DTs against cyberattacks [143,144].
- **Technological update and cost**: The construction of a DT is labor-intensive and demands high-performance computational power, contributing to high investment costs. Additionally, significant knowledge of various technologies (e.g., interactive, edge, ML, and intelligent perception) is required to construct an accurate and real-time DT of a rotating machinery [75,145].

The majority of existing works typically focus on a single component fault or very specific and limited features of interest inside the whole system. However, a DT model should strive towards multi-component faults and interrelated faults. More attention has been paid towards the implementation of a single sensor for generating data for a DT. Approaching the DT via a robust sensor network viewpoint can result in enhancing the accuracy, comprehensiveness, and robustness of DT solutions, even if such networks can pose complexity in knowledge, analysis, interpretation, and computing abilities [146]. Adequate incorporation of the multi-physics approach can particularly influence the DT of rotating machinery significantly, enabling insights about more fault failure scenarios and establishing further suitability towards real-time detecting scenarios. Also, incorporating multiple domains incorporating the full system can improve the scope [147]. Finally, more studies are required to address the difficulties in obtaining real-time data from physical systems (e.g., gearboxes, pumps, structures, and data handling or latency during data transmission).

In summary, this review highlights the potential of an ML-based DT approach, which is more focused on decision making in services (health state monitoring, defect diagnostics, and RUL prediction) and benefits in contrast to the physics-based DT approach that relies on modeling and validating the simulated responses. A summary of the definitions of a DT indicates focus on three main parts: (i) simulation (static feature) and emulation (dynamic feature) for modeling, (ii) data semantics and processing, and (iii) ML-assisted data visualization and optimization for health state monitoring. However, a unified and cohesive concept and an ideal paradigm to establish a DT are still absent. The internal diversity and pathology of a DT, considering the viewpoints and modalities along with the advantages, are subsequently observed. The comprehensive overview of the implementation of DT solutions in transmission and industrial machinery for modeling and characterizing events of interest (health monitoring, anomaly detection, degradation prediction, RUL prediction, etc.) are observed, as well as the challenges encountered and observed performances.

## Figures and Tables

**Table 1:** Consolidation of information extracted from each review article on digital twin applications.

S. No.	Definition of DT	Contribution	Implementing Avenues/Use-Cases	Period and No. of Citations	Focal Points/Tasks	Ref.
1	“The DT consists of a virtual representation of production system that is able to run on different simulation disciplines that is characterized by the synchronization between the virtual and real system, thanks to sensed data and connected smart devices, mathematical models and real time data elaboration”	Paid attention to the practical implementation of DT integration with a control system to monitor production in an assembly line manufacturing execution system.	Manufacturing execution system	2015–2019 (~85)	A DT is constructed to suit a single environment for a laboratory assembly line.	[23]
2	“A set of virtual information constructs that fully describes a potential or actual physical manufactured product from the micro atomic level to the macro geometric level. At its optimum, any information that could be obtained from inspecting a physical manufactured product can be obtained from its digital twin”	Emphasized the significance of AI, ML, and big data in the development of DTs by providing instances from the state-of-the-art deployments already in use for a range of industrial applications.	Manufacturing systems, industrial components, transportation, healthcare, power plants	2015–2020 (~175)	An AI-ML and big-data enabled DT architecture to facilitate a complete DT-enabled system for various industrial avenues is proposed.	[24]
3	“A digital twin is an integrated multi-physics and multi-scale simulation of a product/system that can model the mechanical, electrical, software, and other discipline-specific properties across its life cycle”	Highlighted the use of DT technologies in conjunction with important enabling technologies to advance the design of smart manufacturing systems, which include function, structure, behavior, control, intelligence models, and performance designs.	Manufacturing systems	2014–2020 (~192)	DT technologies implemented towards the design of a smart manufacturing system to assist Industry 4.0 are reviewed.	[25]
4	“A set of adaptive models that emulate the behavior of a physical system in a virtual system getting real time data to update itself along its life cycle. The digital twin replicates the physical system to predict failures and opportunities for changing, to prescribe real time actions for optimizing and/or mitigating unexpected events observing and evaluating the operating profile system”	Attempted to provide a thorough overview of the key elements of DTs and the issues arising from their interactions while highlighting the technical difficulties in designing and developing DTs that are appropriate for a variety of application domains and related technologies.	Manufacturing, aerospace, healthcare, maritime, shipping	2002–2020 (~158)	The features, components, architectures, and functionalities of existing DTs are discussed; however, discussions related to the modularity feature of DTs are not explored.	[26]
5	“A digital twin is a dynamic and self-evolving digital/virtual model or simulation of a real-life subject or object (part, machine, process, human, etc.) representing the exact state of its physical twin at any given point of time via exchanging the real time data as well as keeping the historical data. It is not just the digital twin which mimics its physical twin but any changes in the digital twin are mimicked by the physical twin too”	Intended to consolidate the types of DTs and the definitions of DTs.	NA	2011–2020 (~107)	A brief discussion related to the advantages, classifications, level of integrations, and manifestations of DTs is carried out.	[27]
6	“Digital twin is a kind of simulation that can dynamically mirror the state of a corresponding twin based on the data integrated from the available physical models, sensor updates and operating history, in a multi-physics, multi-scale and probabilistic manner”	Investigated the methods and core technologies (detection, modeling, prognosis, and health management) implemented in the construction of DTs in order to successfully achieve prognostics and health monitoring (PHM) of bearings.	Rolling bearings	2003–2021 (~190)	A comprehensive review regarding the detection, dynamic modeling, and PHM of bearings is performed. The need for the construction of DTs considering the multi-physics coupling and the scope of real, online detection is proliferated briefly.	[9]
7	“DT is a set of virtual information constructs that fully describes a potential or actual physical manufactured product from the micro atomic level to macro geometric level”	Complied and summarized the evolution and developments of DTs and identified the emerging DT types and manifestations for the evolution focus (comprising design, manufacturing, operation, and maintenance) of an electromechanical product life cycle.	Electromechanical products	2011–2022 (~239)	The six emerging core DT technologies distributed and supporting the product life cycle of electromechanical components are discussed.	[28]
8	“Digital twins are digital counterparts of physical entities such as equipment and systems supplied with real-time data that can span from its atomic to geometric level, enabling a more holistic approach to understanding and optimizing the performance of these physical entities”	Reviewed the diverse range of DT predictive models implemented for the fault monitoring (component level and system level) across various industries.	Manufacturing, aviation, transportation, maritime, shipping	2018–2022 (~87)	The exploitation of DT technology for the single fault monitoring of components and systems is reviewed; however, the operating scenarios and simulated/emulated environments need to be disclosed.	[29]
9	“The DT is a representation of an active/unique ‘product’ which can be a real device, object, component, intangible asset, service, or a system consisting of a product and its related services”	Emphasized the application of DTs for streamlining intelligent automation across a range of industrial sectors.	Manufacturing, agriculture, healthcare, aviation, construction	2015–2023 (~92)	The technologies and applications of DTs in various industrial sectors are reviewed briefly.	[5]
10	“The integration of a multidisciplinary, multi-scale simulation process that makes full use of physical models, sensors, operational history, and other data, which serves as a mirror image of the physical product in virtual space and reflects the full life-cycle process of the corresponding physical entity product”	Reviewed the systematic and state-of-the-art approach of a DT assisted with ML algorithms to achieve predictive maintenance, health indicator construction, and RUL prediction.	Civil infrastructure, industrial components	2011–2022 (~125)	The ML-enabled DT architectures proposed to achieve a single component fault diagnosis for various industrial components are reviewed.	[8]
11	“A virtual representation of a physical asset, whose condition is reflected through historical or real-time data captured by sensors”	Focused on the comprehensive review of the applied state-of-the-art DT technologies for the operation and maintenance of an aircraft.	Aircraft	2018–2023 (~72)	The currently implemented DTs for aircraft operation and maintenance are reviewed, and the necessity of an IoT-enabled modular architecture of a DT is highlighted.	[30]
12	“A digital twin is a virtual model duplicating an IoT device’s physical components and behaviors across all phases of its lifespan”	Highlighted the need, process, and features of a DT in Industry 4.0 alongside its major applications.	Industrial applications	2016–2022 (~286)	The applications of DTs for assisting various aspects of Industry 4.0, such as simulating processes, product development, automation, real-time monitoring, better product quality, etc., are reviewed.	[22]

**Table 3:** List of applications of DTs [82].

S. No.	Company	Research Efforts
1	ANSYS	Enhances the physical implementation of various real-time systems through ‘ANSYS twin builder’
2	Bosch	‘Eclipse Ditto’ is an IoT hub which offers communication to various devices over an application programming interface
3	Cisco	Places emphasis on promoting and boosting the performance of a business process through ‘Kinetic IoT platform’
4	Dassault	Optimizes the performance of their products (cars or planes) over its complete life cycle using ‘3DEXPERIENCE’
5	General Electric	Carries out condition monitoring, predicts the performance index, and forecasts the lives of the systems by developing DTs through ‘Predix’
6	IBM	‘DT exchange’ offers technical assistance and cognitive solutions for their clients to develop their industry-centric DTs for the monitoring and prediction of the performance of the systems of their interest
7	Microsoft	Designs and models various environments through ‘Azure DTs’ and uses ‘DT definition language’ to describe the features and events of the DTs
8	Oracle	‘Oracle cloud’ combines the virtual, physical, and augmented reality and IoT knowledge to create DTs for forecasting the condition of various environments
9	Parametric Technology Corporation	Bridges the digital and physical worlds to realize the visualization of innovative products through ‘ThingWorx’
10	Siemens	Focuses on the creation of a smart factory loop in which ‘Siemens Digital Enterprise’ digitalizes the production systems and ‘MindSphere’ develops novel business models

**Table 4:** A summary of some of the published works on the implementation of DT solutions for the health monitoring of various transmission machinery.

Transmission Machinery	Objective	Type of DT	Platform Used	Input Physics Involved	Output Domain Representation	DT Modality	Observations/Remarks	Period and Ref.
Rotor shaft	Fault detection and severity prediction	Analytical	MATLAB/Scilab [105]	Multi-physics (Mechanical + dynamics)	Single domain (Time-domain)	Object twin	The location and progression of the unbalance defect of the rotor shaft are achieved with an error of ~5%.	2019 and [82]
WT gearbox	Anomaly detection	Simulation	MATLAB [105]	Multi-physics (Structural + mechanical + dynamics)	Single domain (Time-domain)	Asset twin	The output torque-based anomaly discrimination is achieved for a three-stage WT transmission system.	2020 and [97]
Toothed belt	Health monitoring	Numerical	Siemens NX MATLAB [105]	Multi-physics (Mechanical + dynamics)	Single domain (Time-domain)	Object twin	The condition of the toothed belt is monitored automatically to enhance the reliability of the machine.	2020 and [106]
Belt drives	Damage identification	Numerical	Abaqus [107]	Multi-physics (Mechanical + dynamics)	Single domain (Time-domain)	Object twin	The combination of parameters causing belt drift is identified.	2021 and [88]
Roller bearing	Fault detection and severity prediction	Analytical	MATLAB [105]	Multi-physics (Mechanical + dynamics)	Single domain (Time-domain and frequency-domain)	Object twin	Through the proposed DT assisted with an ML classifier, five severity levels of bearing outer race defect are classified with an accuracy of 84%.	2021 and [63]
Roller bearing	RUL prediction	Numerical	ANSYS [108]	Multi-physics (Structural + thermal)	Single domain (Time-domain)	Object twin	The RUL prediction of the main bearing with the small datasets is achieved with an accuracy of 85%.	2022 and [92]
Planetary gearbox	Fault diagnosis	Simulation	MATLAB [105]	Multi-physics (Structural + mechanical + dynamics)	Single domain (Time-domain and frequency-domain)	Object twin	The progression of gear tooth degradation is realized.	2022 and [100]
Bush bearing	Fault diagnosis	Analytical	MATLAB [105]	Multi-physics (Mechanical + dynamics)	Single domain (Time-domain)	Object twin	Multi-scale fault (outer race defect) evolution is predicted by examining the vibration response simulated.	2023 and [94]
Production process of shaft	Anomaly detection	Numerical	3D Max Unity [109]	Single physics (Process parameters)	Single domain (Time-domain)	Process twin	Multi-level process parameter monitoring is achieved for identifying the abnormal events in the production process of the transmission shaft.	2023 and [93]
WT gearbox	Monitoring wear progression	Analytical	MATLAB [105]	Multi-physics (Mechanical + dynamics)	Single domain (Time-domain)	Object twin	The gear wear progression is monitored, and the severity of gear tooth degradation of a single-stage spur gearbox is assessed with an accuracy of ~92%.	2023 and [104]
Rotor test rig	Health monitoring	Numerical	SolidWorks 3D Max [109,110]	Multi-physics (Mechanical + thermal + friction)	Multi-domain (Time-domain and rotation)	System twin	Real-time mapping from a physical system to a virtual model is established, and the visual monitoring of the rotor blade rig is achieved.	2023 and [89]

**Table 5:** Summary of some of the published works on the implementation of DT solutions for the health monitoring of various industrial machinery.

Transmission Machinery	Objective	Type of DT	Platform Used	Input Physics Involved	Output Domain Representation	DT Modality	Observations/Remarks	Period and Ref.
Induction motor	Health monitoring	Numerical + simulation	FLUX2D + MATLAB [105,122]	Single physics (Electrical)	Single domain (Time-domain)	Object twin	Waveforms of electromagnetic torque, coil voltage, stator, and rotor current were analyzed for the identification of stator defects of an induction motor from the real-time measurements.	2020 and [117]
Milling machine	Health monitoring	Functional	Java^TM^	Single physics (Structural + mechanical + dynamics)	Multi-domain (Time-domain and delay-domain)	Phenomenon twin	Simulated torque profiles of the milling tool were examined to perform in-process, intelligent health state monitoring.	2020 and [70]
Machine tool	Tool life prediction	Numerical	ANSYS [108]	Multi-physics (Mechanical + thermal + electrical)	Multi-domain (Time-domain and frequency-domain)	Object twin	Simulated tool wear rates were channeled as inputs to the ML algorithms for estimating the RUL progression of a CNC machine tool with an overall error ratio of ~4%.	2020 and [36]
Drilling machine	Health monitoring	Analytical (parametric)	MATLAB/Scilab [105]	Multi-physics (Mechanical + dynamics + electrical)	Single domain (Time-domain)	Object twin	Multi-body dynamical interactions of a drilling machine tool were simulated with an RMS error of ~2%.	2020 and [123]
Machine tool	RUL prediction	Analytical	MATLAB/Scilab [105]	Multi-physics (Mechanical + friction)	Single domain (Time-domain)	Object twin	Degradation parameters (stress, forces, speed, etc.) pertaining to various operating conditions were estimated for the accurate RUL prediction of a CNC machine tool.	2021 and [129]
Triplex pump	Fault diagnosis	Simulation	MATLAB [105]	Multi-physics (Mechanical + thermal)	Single domain (Time-domain)	Asset twin	The pressure signal database was simulated for all the probable defect scenarios of a triplex pump, and fault diagnosis under various operating conditions was achieved with an overall accuracy of ~93%.	2021 and [78]
Induction motor	Health monitoring	Numerical	FEM Magnetics + Python [131]	Multi-physics (Thermal + electromagnetic)	Single domain (Time-domain)	Asset twin	The health state of the machine was monitored by analyzing the simulated electrical conductivity, torque, and temperature profiles.	2022 and [118]
Machine tool	Tool life prediction	Numerical	ANSYS [108]	Multi-physics (Mechanical + hydraulic + electrical)	Single domain (Time-domain)	Phenomenon twin	An evolution model based on fuzzy values to synthesize the evaluation indicators for the successful prediction of the cutting tool life was realized.	2022 and [126]
Machine tool	Fault detection and severity prediction	Numerical	Modelica	Multi-physics (Mechanical + control + electrical)	Single domain (Time-domain)	Object twin	Machine tool spindle stiffness deterioration was assessed with an overall error of ~2%.	2022 and [127]
Triplex pump	Fault diagnosis	Simulation	MATLAB [105]	Multi-physics (Mechanical + thermal)	Single domain (Time-domain)	Asset twin	Intelligent fault diagnosis of the triplex pump subjected to various operating conditions was achieved with an accuracy of ~99%.	2023 and [128]
Induction motor	Anomaly detection	Analytical (phenomenon driven)	MATLAB/Scilab [105]	Single physics (Electrical)	Single domain (Time-domain)	Object twin	Ramanujan periodic transform was implemented on the simulated motor current signature profiles for extracting the features; furthermore, the damage index was constructed for the damage diagnosis of an induction motor.	2023 and [132]
AC synchronous motor	Health monitoring and fault diagnosis	Numerical	FLUX [122]	Multi-physics (Thermal + electromagnetic)	Single domain (Time-domain and frequency-domain)	Asset twin	Through the proposed DT assisted with an ML classifier, eight prominent defects of motor were diagnosed with an overall accuracy of ~94%.	2024 and [121]
Milling machine tool	Condition monitoring	Analytical	MATLAB/Scilab [105]	Multi-physics (Mechanical + friction)	Multi-domain (Time-domain and frequency-domain)	Phenomenon twin	A successful data drive cycle was established among the acquired vibration signals and machine tool control signals through an ML algorithm to predict the machining tool wear rate within the error rate of ~20 μm.	2024 and [133]

## Data Availability

No new data were created or used in this paper while providing the review.

## References

[1] Chatti S., Laperrière L., Reinhart G., Tolio T. (2019). CIRP Encyclopedia of Production Engineering.

[2] Kritzinger W., Karner M., Traar G., Henjes J., Sihn W. (2018). Digital Twin in manufacturing: A categorical literature review and classification. Ifac-PapersOnline.

[3] Minghui H., Ya H., Xinzhi L., Ziyuan L., Jiang Z., Bo M.A. (2023). Digital twin model of gas turbine and its application in warning of performance fault. Chin. J. Aeronaut..

[4] Yang C., Cai B., Wu Q., Wang C., Ge W., Hu Z., Zhu W., Zhang L., Wang L. (2023). Digital twin-driven fault diagnosis method for composite faults by combining virtual and real data. J. Ind. Inf. Integr..

[5] Attaran M., Celik B.G. (2023). Digital Twin: Benefits, use cases, challenges, and opportunities. Decis. Anal. J..

[6] Li X., Shen Y., Cheng H., Yuan F., Huang L. (2022). Identifying the Development Trends and Technological Competition Situations for Digital Twin: A Bibliometric Overview and Patent Landscape Analysis. IEEE Trans. Eng. Manag..

[7] Pantelidakis M., Mykoniatis K., Liu J., Harris G. (2022). A digital twin ecosystem for additive manufacturing using a real-time development platform. Int. J. Adv. Manuf. Technol..

[8] Chen C., Fu H., Zheng Y., Tao F., Liu Y. (2023). The advance of digital twin for predictive maintenance: The role and function of machine learning. J. Manuf. Syst..

[9] Peng F., Zheng L., Peng Y., Fang C., Meng X. (2022). Digital Twin for rolling bearings: A review of current simulation and PHM techniques. Measurement.

[10] Wang Y., Tao F., Zhang M., Wang L., Zuo Y. (2021). Digital twin enhanced fault prediction for the autoclave with insufficient data. J. Manuf. Syst..

[11] Gawde S., Patil S., Kumar S., Kamat P., Kotecha K., Abraham A. (2023). Multi-fault diagnosis of Industrial Rotating Machines using Data-driven approach: A review of two decades of research. Eng. Appl. Artif. Intell..

[12] Vamsi I., Sabareesh G.R., Penumakala P.K. (2019). Comparison of condition monitoring techniques in assessing fault severity for a wind turbine gearbox under non-stationary loading. Mech. Syst. Signal Process..

[13] Moghadam F.K., Nejad A.R. (2022). Online condition monitoring of floating wind turbines drivetrain by means of digital twin. Mech. Syst. Signal Process..

[14] Inturi V., Shreyas N., Chetti K., Sabareesh G.R. (2021). Comprehensive fault diagnostics of wind turbine gearbox through adaptive condition monitoring scheme. Appl. Acoust..

[15] Praveen H.M., Sabareesh G.R., Inturi V., Jaikanth A. (2022). Component level signal segmentation method for multi-component fault detection in a wind turbine gearbox. Measurement.

[16] Inturi V., GR S., Penumakala P.K. (2020). Bearing fault severity analysis on a multi-stage gearbox subjected to fluctuating speeds. Exp. Tech..

[17] Parey A., Singh A. (2019). Gearbox fault diagnosis using acoustic signals, continuous wavelet transform and adaptive neuro-fuzzy inference system. Appl. Acoust..

[18] Pichika S.N., Yadav R., Rajasekharan S.G., Praveen H.M., Inturi V. (2022). Optimal sensor placement for identifying multi-component failures in a wind turbine gearbox using integrated condition monitoring scheme. Appl. Acoust..

[19] Nembhard A.D., Sinha J.K., Pinkerton A.J., Elbhbah K. (2014). Combined vibration and thermal analysis for the condition monitoring of rotating machinery. Struct. Health Monit..

[20] Carroll J., McDonald A., McMillan D. (2016). Failure rate, repair time and unscheduled O&M cost analysis of offshore wind turbines. Wind Energy.

[21] Sheng S., Yang W. Wind turbine drivetrain condition monitoring-an overview (presentation). Proceedings of the 2013 ASME Turbo Expo.

[22] Javaid M., Haleem A. (2023). Digital Twin applications toward Industry 4.0: A Review. Cogn. Robot..

[23] Cimino C., Negri E., Fumagalli L. (2019). Review of digital twin applications in manufacturing. Comput. Ind..

[24] Rathore M.M., Shah S.A., Shukla D., Bentafat E., Bakiras S. (2021). The role of ai, machine learning, and big data in digital twinning: A systematic literature review, challenges, and opportunities. IEEE Access.

[25] Leng J., Wang D., Shen W., Li X., Liu Q., Chen X. (2021). Digital twins-based smart manufacturing system design in Industry 4.0: A review. J. Manuf. Syst..

[26] Semeraro C., Lezoche M., Panetto H., Dassisti M. (2021). Digital twin paradigm: A systematic literature review. Comput. Ind..

[27] Singh M., Fuenmayor E., Hinchy E.P., Qiao Y., Murray N., Devine D. (2021). Digital twin: Origin to future. Appl. Syst. Innov..

[28] Cui Z., Yang X., Yue J., Liu X., Tao W., Xia Q., Wu C. (2023). A review of digital twin technology for electromechanical products: Evolution focus throughout key lifecycle phases. J. Manuf. Syst..

[29] Bofill J., Abisado M., Villaverde J., Sampedro G.A. (2023). Exploring Digital Twin-Based Fault Monitoring: Challenges and Opportunities. Sensors.

[30] Bisanti G.M., Mainetti L., Montanaro T., Patrono L., Sergi I. (2023). Digital twins for aircraft maintenance and operation: A systematic literature review and an IoT-enabled modular architecture. Internet Things.

[31] Wang W., Zhang Y., Gu J., Wang J. (2021). A proactive manufacturing resources assignment method based on production performance prediction for the smart factory. IEEE Trans. Ind. Inform..

[32] Kusiak A. (2018). Smart manufacturing. Int. J. Prod. Res..

[33] Attaran M. (2017). The internet of things: Limitless opportunities for business and society. J. Strateg. Innov. Sustain..

[34] Shu Z., Wan J., Zhang D., Li D. (2016). Cloud-integrated cyber-physical systems for complex industrial applications. Mob. Netw. Appl..

[35] Lv Z., Xie S. (2022). Artificial intelligence in the digital twins: State of the art, challenges, and future research topics. Digit. Twin.

[36] Luo W., Hu T., Ye Y., Zhang C., Wei Y. (2020). A hybrid predictive maintenance approach for CNC machine tool driven by Digital Twin. Robot. Comput. -Integr. Manuf..

[37] Surucu O., Gadsden S.A., Yawney J. (2023). Condition Monitoring using Machine Learning: A Review of Theory, Applications, and Recent Advances. Expert Syst. Appl..

[38] Penchev P., Vitliemov P., Georgiev I. (2023). Optimization model for production scheduling taking into account preventive maintenance in an uncertainty-based production system. Heliyon.

[39] Huang H., Yang L., Wang Y., Xu X., Lu Y. (2021). Digital Twin-driven online anomaly detection for an automation system based on edge intelligence. J. Manuf. Syst..

[40] Inturi V., Penumakala P.K., Sabareesh G.R. (2022). Effect of Multiple Defects and Multi-component Failure on the Dynamic Behaviour of a Wind Turbine Gearbox. Arab. J. Sci. Eng..

[41] Cauchi N., Macek K., Abate A. (2017). Model-based predictive maintenance in building automation systems with user discomfort. Energy.

[42] Chen C., Liu C., Wang T., Zhang A., Wu W., Cheng L. (2023). Compound fault diagnosis for industrial robots based on dual-transformer networks. J. Manuf. Syst..

[43] Inturi V., Balaji S.V., Gyanam P., Pragada B.P.V., Geetha Rajasekharan S., Pakrashi V. (2023). An integrated condition monitoring scheme for health state identification of a multi-stage gearbox through Hurst exponent estimates. Struct. Health Monit..

[44] Alzghoul A., Backe B., Löfstrand M., Byström A., Liljedahl B. (2014). Comparing a knowledge-based and a data-driven method in querying data streams for system fault detection: A hydraulic drive system application. Comput. Ind..

[45] Liu Z., Chen W., Zhang C., Yang C., Chu H. (2019). Data super-network fault prediction model and maintenance strategy for mechanical product based on digital twin. IEEE Access.

[46] Glaessgen E., Stargel D. The digital twin paradigm for future NASA and US Air Force vehicles. Proceedings of the 53rd AIAA/ASME/ASCE/AHS/ASC Structures, Structural Dynamics and Materials Conference 20th AIAA/ASME/AHS Adaptive Structures Conference 14th AIAA.

[47] Liu J., Liu X., Vatn J., Yin S. (2023). A generic framework for qualifications of digital twins in maintenance. J. Autom. Intell..

[48] Schleich B., Anwer N., Mathieu L., Wartzack S. (2017). Shaping the digital twin for design and production engineering. CIRP Ann..

[49] Awouda A., Traini E., Bruno G., Chiabert P. (2024). IoT-Based Framework for Digital Twins in the Industry 5.0 Era. Sensors.

[50] Papacharalampopoulos A., Foteinopoulos P., Stavropoulos P. (2023). Integration of Industry 5.0 requirements in digital twin-supported manufacturing process selection: A framework. Procedia CIRP.

[51] Soori M., Arezoo B., Dastres R. (2023). Digital Twin for Smart Manufacturing, A Review. Sustain. Manuf. Serv. Econ..

[52] Emmert-Streib F. (2023). Defining a Digital Twin: A Data Science-Based Unification. Mach. Learn. Knowl. Extr..

[53] Ayani M., Ganebäck M., Ng A.H. (2018). Digital Twin: Applying emulation for machine reconditioning. Procedia CIRP.

[54] You Y., Chen C., Hu F., Liu Y., Ji Z. (2022). Advances of digital twins for predictive maintenance. Procedia Comput. Sci..

[55] Boschert S., Rosen R. (2016). Digital twin—The simulation aspect. Mechatronic Futures.

[56] Zhao W., Zhang C., Fan B., Wang J., Gu F., Peyrano O.G., Wang S., Lv D. (2023). Research on rolling bearing virtual-real fusion life prediction with digital twin. Mech. Syst. Signal Process..

[57] Yang X., Ran Y., Zhang G., Wang H., Mu Z., Zhi S. (2022). A digital twin-driven hybrid approach for the prediction of performance degradation in transmission unit of CNC machine tool. Robot. Comput. Integr. Manuf..

[58] He R., Chen G., Dong C., Sun S., Shen X. (2019). Data-driven digital twin technology for optimized control in process systems. ISA Trans..

[59] Cai Y., Starly B., Cohen P., Lee Y.S. (2017). Sensor data and information fusion to construct digital-twins virtual machine tools for cyber-physical manufacturing. Procedia Manuf..

[60] Melesse T.Y., Di Pasquale V., Riemma S. (2021). Digital Twin models in industrial operations: State-of-the-art and future research directions. IET Collab. Intell. Manuf..

[61] Abramovici M., Göbel J.C., Savarino P. (2017). Reconfiguration of smart products during their use phase based on virtual product twins. CIRP Ann..

[62] Yu G., Wang Y., Mao Z., Hu M., Sugumaran V., Wang Y.K. (2021). A digital twin-based decision analysis framework for operation and maintenance of tunnels. Tunn. Undergr. Space Technol..

[63] Farhat M.H., Chiementin X., Chaari F., Bolaers F., Haddar M. (2021). Digital twin-driven machine learning: Ball bearings fault severity classification. Meas. Sci. Technol..

[64] Zhong D., Xia Z., Zhu Y., Duan J. (2023). Overview of predictive maintenance based on digital twin technology. Heliyon.

[65] Inturi V., Shreyas N., Sabareesh G.R. (2021). Anfis-based defect severity prediction on a multi-stage gearbox operating under fluctuating speeds. Neural Process. Lett..

[66] Praveen H.M., Jaikanth A., Inturi V., Sabareesh G.R. (2021). Fingerprinting based data abstraction technique for remaining useful life estimation in a multi-stage gearbox. Measurement.

[67] Botín-Sanabria D.M., Mihaita A.S., Peimbert-García R.E., Ramírez-Moreno M.A., Ramírez-Mendoza R.A., Lozoya-Santos J.D.J. (2022). Digital twin technology challenges and applications: A comprehensive review. Remote Sens..

[68] Ladj A., Wang Z., Meski O., Belkadi F., Ritou M., Da Cunha C. (2021). A knowledge-based Digital Shadow for machining industry in a Digital Twin perspective. J. Manuf. Syst..

[69] Calvo-Bascones P., Voisin A., Do P., Sanz-Bobi M.A. (2023). A collaborative network of digital twins for anomaly detection applications of complex systems. Snitch Digital Twin concept. Comput. Ind..

[70] Ghosh A.K., Ullah A.S., Kubo A., Akamatsu T., D’Addona D.M. (2020). Machining phenomenon twin construction for industry 4.0: A case of surface roughness. J. Manuf. Mater. Process..

[71] Li Y., Tao Z., Wang L., Du B., Guo J., Pang S. (2023). Digital twin-based job shop anomaly detection and dynamic scheduling. Robot. Comput. Integr. Manuf..

[72] Redelinghuys A.J.H., Basson A.H., Kruger K. (2020). A six-layer architecture for the digital twin: A manufacturing case study implementation. J. Intell. Manuf..

[73] Xia P., Huang Y., Tao Z., Liu C., Liu J. (2023). A digital twin-enhanced semi-supervised framework for motor fault diagnosis based on phase-contrastive current dot pattern. Reliab. Eng. Syst. Saf..

[74] Liu Q., Zhang H., Leng J., Chen X. (2019). Digital twin-driven rapid individualised designing of automated flow-shop manufacturing system. Int. J. Prod. Res..

[75] Tao F., Cheng J., Qi Q., Zhang M., Zhang H., Sui F. (2018). Digital twin-driven product design, manufacturing and service with big data. Int. J. Adv. Manuf. Technol..

[76] Chakraborty S., Adhikari S., Ganguli R. (2021). The role of surrogate models in the development of digital twins of dynamic systems. Appl. Math. Model..

[77] Grieves M., Vickers J. (2017). Digital twin: Mitigating unpredictable, undesirable emergent behavior in complex systems. Transdisciplinary Perspectives on Complex Systems: New Findings and Approaches.

[78] Xia M., Shao H., Williams D., Lu S., Shu L., de Silva C.W. (2021). Intelligent fault diagnosis of machinery using digital twin-assisted deep transfer learning. Reliab. Eng. Syst. Saf..

[79] Shen W., Yang C., Gao L. (2020). Address business crisis caused by COVID-19 with collaborative intelligent manufacturing technologies. IET Collab. Intell. Manuf..

[80] (2020). Gartner Survey Reveals 47% of Organizations Will Increase Investments in IoT Despite the Impact of COVID-19. https://www.gartner.com/en/newsroom/press-releases/2020-10-29-gartner-survey-reveals-47-percent-of-organizations-will-increase-investments-in-iot-despite-the-impact-of-covid-19-.

[81] Ma J., Chen H., Zhang Y., Guo H., Ren Y., Mo R., Liu L. (2020). A digital twin-driven production management system for production workshop. Int. J. Adv. Manuf. Technol..

[82] Wang J., Ye L., Gao R.X., Li C., Zhang L. (2019). Digital Twin for rotating machinery fault diagnosis in smart manufacturing. Int. J. Prod. Res..

[83] Chakraborty S., Adhikari S. (2021). Machine learning based digital twin for dynamical systems with multiple time-scales. Comput. Struct..

[84] Garg S., Gogoi A., Chakraborty S., Hazra B. (2021). Machine learning based digital twin for stochastic nonlinear multi-degree of freedom dynamical system. Probabilistic Eng. Mech..

[85] Ritto T.G., Rochinha F.A. (2021). Digital twin, physics-based model, and machine learning applied to damage detection in structures. Mech. Syst. Signal Process..

[86] Wang M., Wang C., Hnydiuk-Stefan A., Feng S., Atilla I., Li Z. (2021). Recent progress on reliability analysis of offshore wind turbine support structures considering digital twin solutions. Ocean Eng..

[87] Yu J., Song Y., Tang D., Dai J. (2021). A Digital Twin approach based on nonparametric Bayesian network for complex system health monitoring. J. Manuf. Syst..

[88] Fedorko G., Molnar V., Vasiľ M., Salai R. (2021). Proposal of digital twin for testing and measuring of transport belts for pipe conveyors within the concept Industry 4.0. Measurement.

[89] Duan J.G., Ma T.Y., Zhang Q.L., Liu Z., Qin J.Y. (2023). Design and application of digital twin system for the blade-rotor test rig. J. Intell. Manuf..

[90] Liu C., Ricardo Mauricio A., Qi J., Peng D., Gryllias K. (2020). Domain adaptation digital twin for rolling element bearing prognostics. Annu. Conf. PHM Soc..

[91] Piltan F., Kim J.M. (2021). Crack size identification for bearings using an adaptive digital twin. Sensors.

[92] Zhao W., Zhang C., Wang J., Peyrano O.G., Gu F., Wang S., Lv D. (2022). Research on main bearing life prediction of direct-drive wind turbine based on digital twin technology. Meas. Sci. Technol..

[93] Li T., Shi H., Bai X., Zhang K. (2023). A Digital Twin Model of Life-Cycle Rolling Bearing With Multiscale Fault Evolution Combined With Different Scale Local Fault Extension Mechanism. IEEE Trans. Instrum. Meas..

[94] Regis A., Arroyave-Tobón S., Linares J.M., Mermoz E. (2023). Physic-based vs data-based digital twins for bush bearing wear diagnostic. Wear.

[95] Zhang Y., Ji J.C., Ren Z., Ni Q., Gu F., Feng K., Yu K., Ge J., Lei Z., Liu Z. (2023). Digital twin-driven partial domain adaptation network for intelligent fault diagnosis of rolling bearing. Reliab. Eng. Syst. Saf..

[96] Shi H., Song Z., Bai X., Hu Y., Li T., Zhang K. (2023). A novel digital twin model for dynamical updating and real-time mapping of local defect extension in rolling bearings. Mech. Syst. Signal Process..

[97] Xiangjun Z., Ming Y., Xianglong Y., Yifan B., Chen F., Yu Z. Anomaly detection of wind turbine gearbox based on digital twin drive. Proceedings of the 2020 IEEE 3rd Student Conference on Electrical Machines and Systems (SCEMS).

[98] Hu J., Hu N., Luo P., Yang Y. Fault Diagnosis of Gearbox Based on Digital Twin Concept Model. Proceedings of the 2021 4th International Conference on Intelligent Robotics and Control Engineering (IRCE).

[99] Fahim M., Sharma V., Cao T.V., Canberk B., Duong T.Q. (2022). Machine learning-based digital twin for predictive modeling in wind turbines. IEEE Access.

[100] Zhu D., Li Z., Hu N. (2022). Multi-Body Dynamics Modeling and Analysis of Planetary Gearbox Combination Failure Based on Digital Twin. Appl. Sci..

[101] Mehlan F.C., Pedersen E., Nejad A.R. (2022). Modelling of wind turbine gear stages for digital twin and real-time virtual sensing using bond graphs. J. Phys. Conf. Ser..

[102] Yu J., Wang S., Wang L., Sun Y. (2023). Gearbox fault diagnosis based on a fusion model of virtual physical model and data-driven method. Mech. Syst. Signal Process..

[103] Wang Y., Sun W., Liu L., Wang B., Bao S., Jiang R. (2023). Fault Diagnosis of Wind Turbine Planetary Gear Based on a Digital Twin. Appl. Sci..

[104] Feng K., Ji J.C., Zhang Y., Ni Q., Liu Z., Beer M. (2023). Digital twin-driven intelligent assessment of gear surface degradation. Mech. Syst. Signal Process..

[105] The MathWorks, Inc. (R2023b). https://in.mathworks.com/products/matlab-home.html.

[106] Malaka J., Hetmańczyk M. (2020). Intelligent Drive in Industry 4.0–Protection of Toothed Belt Transmission on the Basis of Its Digital Twin. Sympozjon Modelowanie w Mechanice.

[107] Abaqus Unified FEA–SIMULIA by Dassault Systems. https://www.3ds.com/products/simulia.

[108] Ansys, Inc. https://www.ansys.com/en-in.

[109] Unity Software Inc. https://unity.com/.

[110] Solidworks. https://www.solidworks.com/.

[111] Adamou A.A., Alaoui C. (2023). Energy efficiency model-based Digital shadow for Induction motors: Towards the implementation of a Digital Twin. Eng. Sci. Technol. Int. J..

[112] Söderäng E., Hautala S., Mikulski M., Storm X., Niemi S. (2022). Development of a digital twin for real-time simulation of a combustion engine-based power plant with battery storage and grid coupling. Energy Convers. Manag..

[113] Mertes J., Glatt M., Schellenberger C., Klar M., Schotten H.D., Aurich J.C. (2022). Development of a 5G-enabled Digital Twin of a Machine Tool. Procedia CIRP.

[114] Nguyen T.N., Ponciroli R., Bruck P., Esselman T.C., Rigatti J.A., Vilim R.B. (2022). A digital twin approach to system-level fault detection and diagnosis for improved equipment health monitoring. Ann. Nucl. Energy.

[115] Tshoombe B.K., Dos Santos J.F., Araújo R.C., Fonseca W.D.S. Implementation of DT-based monitoring system of induction motors. Proceedings of the 2021 14th IEEE International Conference on Industry Applications (INDUSCON).

[116] Bondarenko O., Fukuda T. (2020). Development of a diesel engine’s digital twin for predicting propulsion system dynamics. Energy.

[117] Bouzid S., Viarouge P., Cros J. (2020). Real-time digital twin of a wound rotor induction machine based on finite element method. Energies.

[118] Dos Santos J.F., Tshoombe B.K., Santos L.H., Araújo R.C., Manito A.R., Fonseca W.S., Silva M.O. (2022). Digital Twin-Based Monitoring System of Induction Motors Using IoT Sensors and Thermo-Magnetic Finite Element Analysis. IEEE Access.

[119] Huang Y., Yuan B., Xu S., Han T. (2022). Fault Diagnosis of Permanent Magnet Synchronous Motor of Coal Mine Belt Conveyor Based on Digital Twin and ISSA-RF. Processes.

[120] Wang J., Zhang Z., Liu Z., Han B., Bao H., Ji S. (2023). Digital twin aided adversarial transfer learning method for domain adaptation fault diagnosis. Reliab. Eng. Syst. Saf..

[121] Kohtz S., Zhao J., Renteria A., Lalwani A., Xu Y., Zhang X., Haran K.S., Senesky D., Wang P. (2024). Optimal sensor placement for permanent magnet synchronous motor condition monitoring using a digital twin-assisted fault diagnosis approach. Reliab. Eng. Syst. Saf..

[122] Altair Engineering Inc. https://altair.com/flux.

[123] Wang C.P., Erkorkmaz K., McPhee J., Engin S. (2020). In-process digital twin estimation for high-performance machine tools with coupled multibody dynamics. CIRP Ann..

[124] Ghosh A.K., Ullah A.S., Teti R., Kubo A. (2021). Developing sensor signal-based digital twins for intelligent machine tools. J. Ind. Inf. Integr..

[125] Zhu Z., Xi X., Xu X., Cai Y. (2021). Digital Twin-driven machining process for thin-walled part manufacturing. J. Manuf. Syst..

[126] Wei Y., Hu T., Wei S., Ma S., Wang Y. (2024). Digital twin technology applicability evaluation method for CNC machine tool. Int. J. Adv. Manuf. Technol..

[127] Xue R., Zhang P., Huang Z., Wang J. (2024). Digital twin-driven fault diagnosis for CNC machine tool. Int. J. Adv. Manuf. Technol..

[128] Wang J., Niu X., Gao R.X., Huang Z., Xue R. (2023). Digital twin-driven virtual commissioning of machine tool. Robot. Comput. -Integr. Manuf..

[129] Guo J., Yang Z., Chen C., Luo W., Hu W. (2021). Real-time prediction of remaining useful life and preventive maintenance strategy based on digital twin. J. Comput. Inf. Sci. Eng..

[130] Finite Element Method Magnetics. https://www.femm.info/wiki/HomePage.

[131] Liu Z., Chen W., Zhang C., Yang C., Cheng Q. (2021). Intelligent scheduling of a feature-process-machine tool supernetwork based on digital twin workshop. J. Manuf. Syst..

[132] Hu W., Wang T., Chu F. (2023). A novel Ramanujan digital twin for motor periodic fault monitoring and detection. IEEE Trans. Ind. Inform..

[133] Liu Z., Lang Z.Q., Gui Y., Zhu Y.P., Laalej H. (2024). Digital twin-based anomaly detection for real-time tool condition monitoring in machining. J. Manuf. Syst..

[134] Guerra-Zubiaga D., Kuts V., Mahmood K., Bondar A., Nasajpour-Esfahani N., Otto T. (2021). An approach to develop a digital twin for industry 4.0 systems: Manufacturing automation case studies. Int. J. Comput. Integr. Manuf..

[135] Li T.J., Wang M.Z., Zhao C.Y. (2021). Study on real-time thermal–mechanical–frictional coupling characteristics of ball bearings based on the inverse thermal network method. Proc. Inst. Mech. Eng. Part J J. Eng. Tribol..

[136] Ye Z., Wang L., Chen G., Tang D. (2017). Analysis of thermo-mechanical coupling of high-speed angular-contact ball bearings. Adv. Mech. Eng..

[137] He B., Bai K.J. (2021). Digital twin-based sustainable intelligent manufacturing: A review. Adv. Manuf..

[138] Adamenko D., Kunnen S., Pluhnau R., Loibl A., Nagarajah A. (2020). Review and comparison of the methods of designing the Digital Twin. Procedia CIRP.

[139] Zhao C., Shen W. (2022). Dual adversarial network for cross-domain open set fault diagnosis. Reliab. Eng. Syst. Saf..

[140] Sittón-Candanedo I., Alonso R.S., Corchado J.M., Rodríguez-González S., Casado-Vara R. (2019). A review of edge computing reference architectures and a new global edge proposal. Future Gener. Comput. Syst..

[141] Shao G., Helu M. (2020). Framework for a digital twin in manufacturing: Scope and requirements. Manuf. Lett..

[142] Wagner R., Schleich B., Haefner B., Kuhnle A., Wartzack S., Lanza G. (2019). Challenges and potentials of digital twins and industry 4.0 in product design and production for high performance products. Procedia CIRP.

[143] Falekas G., Karlis A. (2021). Digital twin in electrical machine control and predictive maintenance: State-of-the-art and future prospects. Energies.

[144] Khan S., Farnsworth M., McWilliam R., Erkoyuncu J. (2020). On the requirements of digital twin-driven autonomous maintenance. Annu. Rev. Control.

[145] Krüger S., Borsato M. (2019). Developing knowledge on digital manufacturing to digital twin: A bibliometric and systemic analysis. Procedia Manuf..

[146] Sleiti A.K., Kapat J.S., Vesely L. (2022). Digital twin in energy industry: Proposed robust digital twin for power plant and other complex capital-intensive large engineering systems. Energy Rep..

[147] de Wilde P. (2023). Building performance simulation in the brave new world of artificial intelligence and digital twins: A systematic review. Energy Build..

